# Mismatched online public concern and tick-borne disease risk in China

**DOI:** 10.1016/j.soh.2024.100101

**Published:** 2024-12-12

**Authors:** Yuxin Li, Tengfei Hu, Tingting Wang, Sen Li

**Affiliations:** a School of Environmental Science and Engineering, Huazhong University of Science and Technology, Wuhan 430074, Hubei, China; b School of Art Design and Media, Wuhan Huaxia Institute of Technology, Wuhan 430223, Hubei, China

**Keywords:** Ticks, Tick-borne diseases, Public awareness, Public opinion data, China

## Abstract

**Introduction:**

Ticks and tick-borne diseases are increasing public health concerns in China. This study examines public awareness and concern using data from the People Cloud, a national public opinion data platform.

**Methods:**

We analyzed 358,862 posts related to ticks, tick bites, Lyme disease, and tick-borne encephalitis from October 2022 to November 2023. Temporal trends and geographic distribution were assessed to identify patterns of public interest.

**Results:**

Public concern peaked in April, coinciding with tick season, with “ticks” being the most mentioned term, particularly on social media platforms, such as WeChat. High search activity was concentrated in southeastern and northern provinces, especially Zhejiang, Guangdong, and Jiangsu. Lyme disease posts were more dispersed, while tick-borne encephalitis posts clustered in southern areas.

**Conclusion:**

Public interest in ticks follows seasonal and regional trends, reflecting tick activity but not always matching disease risk or tick distribution. Monitoring public opinion data can guide targeted health interventions and improve disease prevention efforts.

## 1 Introduction

Ticks and tick-borne diseases have emerged as increasing public health concerns globally, with rising cases reported in various regions, including China [1]. Ticks act as vectors for numerous pathogens, transmitting diseases such as Lyme disease, tick-borne encephalitis, and other illnesses that threaten the health of both humans and livestock [2]. The risks posed by these vector-borne zoonotic diseases carry significant implications for public health [3], particularly in regions where outdoor activities are common, and human–tick interaction is more likely.

In recent years, public awareness and concern about ticks and tick-borne diseases has risen, largely driven by media coverage and public health campaigns. Understanding public perception of these threats, along with their awareness levels, offers valuable insights for public health officials and policymakers. Traditional methods of monitoring public concern, such as surveys and targeted investigations [4,5], are valuable, yet often constrained by time and geographic limitations. These methods often fail to capture real-time shifts in public sentiment or overlook important regional differences. Big data platforms, capable of collecting and analyzing public opinion in a timely manner, present an innovative solution for assessing these concerns in real-time. For example, several studies in Italy and Germany have employed Google Trends to monitor public interest in tick-borne diseases, showing a significant correlation between internet search data and incidences of infectious diseases [6,7]. However, the effectiveness of such platforms relies on their ability to adapt to local contexts and data sources.

Public concern about tick-borne diseases in China is driven by rising case numbers [8], expanding ecotourism [9], media coverage [10], and environmental changes [11]. To address these concerns effectively, public health efforts should focus on prevention, education, and control. Analyzing search terms and social media posts related to ticks can provide valuable insights into the timing and location of public concern and the geographic distribution of discussion [7]. This can help researchers and policymakers identify disease patterns, assess awareness gaps, and improve efforts to mitigate tick-borne disease risks. While tools like Google Trends offer insights into public perception, they have notable limitations in China. They primarily track search activity, which may not fully capture concern across diverse demographic groups or regions with varying internet use. Additionally, these tools rely on active searches, excluding those less engaged online or using platforms like WeChat or Weibo, which are more popular in China.

This study aims to investigate public concerns about ticks and tick-borne diseases in China by analyzing data from the People Cloud platform, an intelligent public opinion management tool based on big data mining technology. People Cloud enables comprehensive analysis of public discourse across multiple regions and provides more accurate insights by capturing a broader range of public discourse, including posts from popular Chinese platforms such as WeChat and Weibo. Specifically, the study will examine keyword search volumes and posts related to ticks, tick bites, Lyme disease, and tick-borne encephalitis to identify trends in public awareness and the geographic distribution of interest.

## 2 Methods

### 2.1 Study design

This study employed a descriptive design to investigate public concerns regarding ticks and tick-borne diseases in China, utilizing data from People Cloud (https://rmzy.People.Cloud.cn/), a standardized SaaS (Software as a Service) public opinion service platform developed by the People's Daily Online Public Opinion Data Center/People Online (https://www.peopleonline.cn/), which leverages advanced big data technology and extensive experience in public opinion services. The platform's keyword search tool was employed to track the frequency of posts related to ticks and tick-borne diseases. The search focused on four primary keywords (in Chinese): "ticks," "tick bite," "Lyme disease," and "tick-borne encephalitis." These keywords represent different facets of public concern: “ticks” refers to general discussions about ticks, while “tick bite” focuses on direct human–tick interactions. This differentiation helps in understanding the specific areas of public interest and concern. The study included only users with China-based IP addresses, ensuring that the data accurately reflected domestic concerns. This approach facilitated a comprehensive exploration of keyword trends over time and across geographic regions.

### 2.2 Data collection

Data were collected using People Cloud's keyword search feature, which provided post volumes related to specific keywords. This involved entering each keyword into the platform's search tool, returning relevant post volumes for the specified time frame. In this study, “posts” are defined as individual pieces of content retrieved from various online platforms, including social media updates, articles, blog entries, comments, and videos, as aggregated by the People Cloud platform. The primary data sources included WeChat, Sina Weibo, and news aggregation sites. WeChat, China's largest instant messaging app with over 1 billion users, facilitates information sharing through features like Moments, Official Accounts, and Group Chats. Sources on WeChat included user-generated content and articles from Official Accounts. Sina Weibo functions similarly to Twitter, allowing short posts, images, and videos, with a strong youth user base contributing user posts and trending hashtags. Today's Headlines (Toutiao) is an algorithm-driven news platform that curates content based on user interests, sourcing from creator publications and user feedback. We selected the period from October 2022 to November 2023 to capture the most recent full year of data, encompassing all seasons of tick activity. This allowed for an analysis of current public interest and seasonal patterns, providing timely insights for public health responses.

To ensure data reliability, we cross-validated the post volumes obtained from People Cloud with data from individual platforms where possible. In evaluating post quality, we applied a content analysis framework to identify incomplete, biased, or equivocal information. Posts were categorized based on clarity, source credibility, and relevance to tick-borne diseases. Irrelevant or unreliable posts were excluded to minimize bias, ensuring that the analysis reflects genuine public concern and awareness rather than misinformation. The search results included total keyword mentions and geographic data based on user locations. To ensure comprehensive analysis, the study focused on posts from across China using the aforementioned four keywords to assess public interest and concern. The data were aggregated monthly to analyze trends over time and by province to map the spatial distribution of posts.

### 2.3 Spatio-temporal analysis

The number of posts for each keyword was recorded and summarized to visualize temporal trends. Monthly keyword usage was analyzed to identify peak periods of public interest. Additionally, the data were plotted to visualize the spatial distribution of tick-related posts across Chinese provincial level administration divisions, enabling the assessment of regions with the highest levels of public concern and the geographic clustering of tick-related discussions.

To assess the disparity between the geographic distribution of tick activity and public concern, we compared tick occurrence data (summarized from a recent study [12]) with the volume of online posts at the provincial level. Both datasets were normalized to account for differences in reporting frequency and population size across provinces. A scatter plot was then generated to visually represent the disparity between tick activity and online posts. This comparison allowed us to identify regions with notable mismatches, such as areas with high online concern but low tick activity, providing insights into potential gaps in public awareness or the need for targeted education and outreach efforts.

To further investigate the discrepancies between tick occurrence and public concern, we employed Bivariate Moran's Ⅰ, a spatial statistic that measures the degree of spatial autocorrelation between two variables. This method identifies clusters of areas where similar values for both variables (e.g., high tick occurrence and high public concern) are spatially grouped. The resulting value ranges from −1 (negative spatial autocorrelation) to +1 (positive spatial autocorrelation), with 0 indicating no spatial autocorrelation. Positive values suggest that areas with similar values for both variables are clustered (e.g., high tick occurrence and high public concern), while negative values indicate a mismatch (e.g., high tick occurrence and low public concern). Based on these values, we identified four clusters: high–high (high tick occurrence and high concern), low–low (low tick occurrence and low concern), low-high (low tick occurrence but high concern), and high-low (high tick occurrence but low concern), providing insights into the spatial patterns between tick-borne disease risk and public awareness. Geographic patterns were examined, heatmaps were generated, and Bivariate Moran's Ⅰ were calculated using *ArcMap* (v10.8.1) and *GeoDa* (v1.22) software.

## 3 Results

### 3.1 Public concern over ticks dominates online discussions

From October 2022 to November 2023, the People Cloud platform identified a total of 358,862 posts related to ticks and tick-borne diseases across China. The most frequently searched keyword was “ticks”, accounting for 53.15 % (*n* = 190,738) of all posts. This was followed by “tick bite”, which comprised 34.12 % (*n* = 122,447) of the total post volume. Disease-specific keywords, such as “Lyme disease” and “tick-borne encephalitis”, represented smaller proportions, with 8.06 % (*n* = 28,924) and 4.67 % (*n* = 16,753) of the posts, respectively.

Table 1 presents records for different keywords across various platforms. “ticks” emerges as the most mentioned term, particularly on WeChat, indicating strong public interest. Other frequently discussed keywords include “tick bites”, “Lyme disease”, and “tick-borne encephalitis” with mentions decreasing in frequency. WeChat and Weibo dominate the discussions, while engagement from government institutions and digital newspapers remains significantly lower. Overall, the data suggests a considerable public focus on tick-related health issues, especially within social media channels.

**Table 1:** Distribution of posts across various online platforms for each keyword.

Keywords	WeChat	Weibo	News	Network video	Network media	Forum	Digital newspaper	Government institutions
Ticks	73,832	50,531	29,423	7831	2929	2065	609	463
Tick bites	63,177	21,803	21,017	519	1947	1009	395	278
Lyme disease	9856	7920	6052	112	484	426	82	112
Tick-borne encephalitis	9787	711	3177	68	457	309	94	131

### 3.2 Seasonal surge in tick-related posts peaks in April

**Fig. 1:**
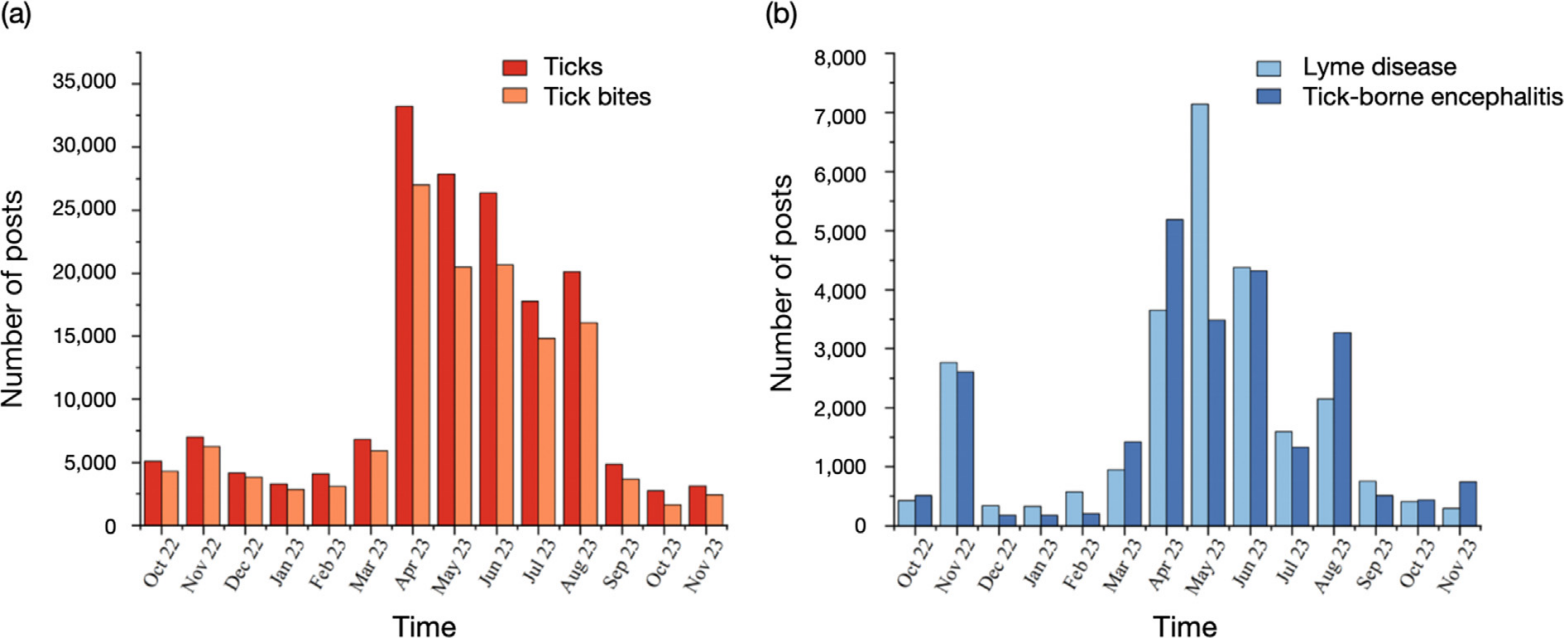
Seasonal changes in tick-related online posts in China.

The data revealed a distinct seasonal pattern in the volume of tick-related posts. A sharp increase in search volume began in March 2023, continuing to rise and peaking in April 2023 (Fig. 1). The highest number of posts in a single month occurred in April 2023, totaling 33,224 posts related to ticks and tick-borne diseases. After this peak, post volume gradually declined throughout the summer and early autumn months.

Both “ticks” and “tick bite” exhibited a similar temporal pattern, peaking in April 2023, which is at 33,224 and 27,015, respectively (Fig. 1a). “Ticks” consistently maintained higher search volumes compared to “tick bite” throughout the study period. Keywords related to diseases also demonstrated a similar seasonal trend. Posts mentioning “forest encephalitis” peaked in April 2023 (*n* = 7138), whereas “Lyme disease” reached its highest post volume in May 2023 (*n* = 5187) (Fig. 1b). This suggests a correlation between public concern and the typical tick season, which generally spans spring through autumn.

### 3.3 Southeastern and northern China lead in public concern

**Fig. 2:**
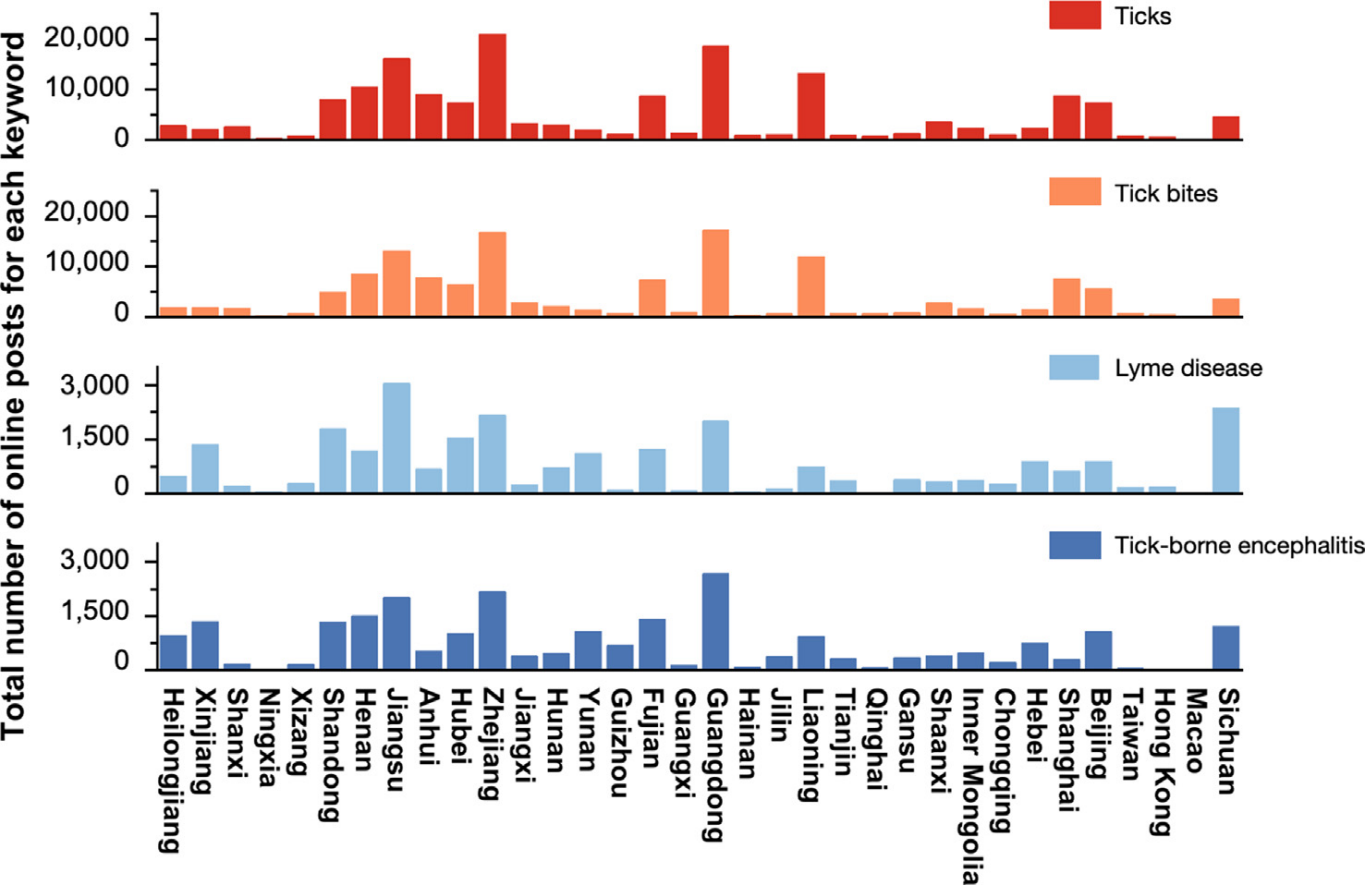
Geographic distribution of tick-related online posts in China.

The spatial distribution of posts varied across provinces (Fig. 2), with the majority of tick-related keyword searches concentrated in southeastern and northern China. Zhejiang (*n* = 20,926), a southeastern coastal province, Guangdong (*n* = 18,583), located in the south near Hong Kong Special Administrative Region, Jiangsu (*n* = 16,084), on the eastern coast, and Liaoning (*n* = 13,230), in the northeast, had the highest number of posts mentioning “ticks.” These provinces also exhibited high search volumes for “tick bite,” displaying similar geographic clustering.

In contrast, the geographic distribution of posts related to tick-borne diseases revealed some variations. Lyme disease was more widely distributed across northern and southern provinces. The highest number of posts related to Lyme disease was observed in Jiangsu (*n* = 3048), followed by Sichuan (in the southwest), Zhejiang, Guangdong, Shandong (in northern China), and Hubei (in central China). Posts mentioning “tick-borne encephalitis” were primarily concentrated in Guangdong (*n* = 2669), followed by Zhejiang, Jiangsu, Henan (a northern inland province), and Fujian (another southeastern coastal province).

The distribution of posts related to “ticks” and “tick bites” reveals notable concentrations in southeastern coastal provinces, such as Zhejiang and Guangdong, as well as in certain northern regions like Liaoning and Heilongjiang. An analysis of the total number of posts compared to per capita posts highlights distinct patterns (Supplemental Figure S1). Notably, Xizang Autonomous Region and Qinghai show relatively low total post counts, which sharply contrasts with their per capita statistics. In examining the distribution of “Lyme disease” and “tick-borne encephalitis”, Xizang Autonomous Region displays a consistent pattern in both total and per capita posts. The primary areas of interest regarding ticks are concentrated in Xizang Autonomous Region, Zhejiang, and Liaoning. In contrast, discussions surrounding Lyme disease are predominantly centered in Xinjiang Uygur Autonomous Region, Xizang Autonomous Region, and Yunnan, while significant posts about tick-borne encephalitis primarily originate from Xinjiang Uygur Autonomous Region and Xizang Autonomous Region.

### 3.4 Spatial disparities in tick occurrence and public concern

We assessed the disparity between the geographic distribution of tick activity and the volume of online posts to identify regional mismatches, revealing significant variations in the alignment between public concern and actual tick occurrence. Specifically, Zhejiang, Jiangsu, and Guangdong had high levels of online concern about ticks, despite relatively low recorded tick activity, while Xinjiang Uygur Autonomous Region, Shaanxi, and Heilongjiang exhibited high tick occurrence but lower levels of online concern.

**Fig. 3:**
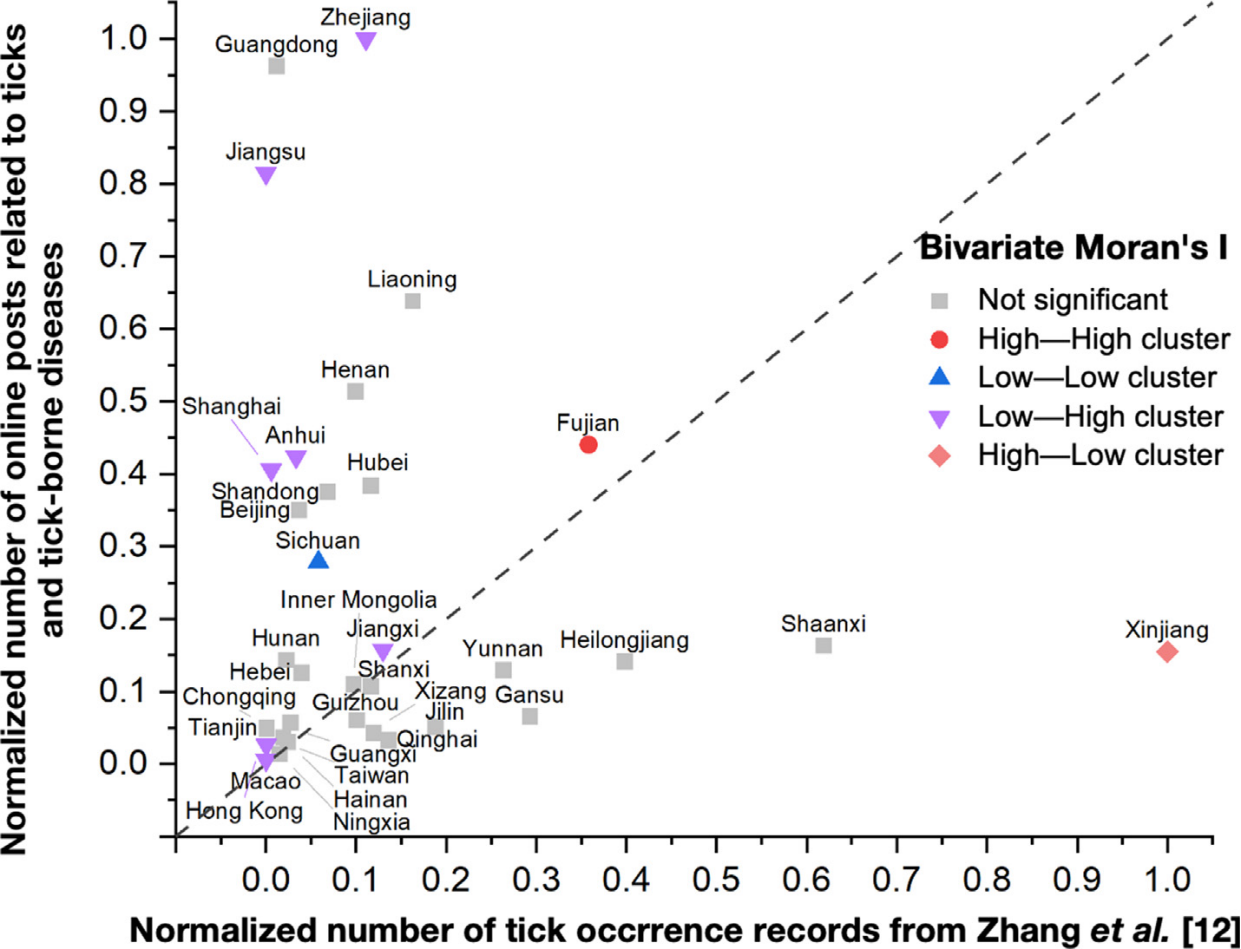
Spatial distribution of tick occurrence and public concern across provinces. Tick occurrence data were compiled from a point-based dataset [12], which includes literature reports on tick species from 1960 to 2017.

Bivariate Moran's Ⅰ analysis identified notable spatial clusters of high and low tick occurrence and public concern. Regions like Fujian exhibited both high tick activity and high public concern (high–high cluster), while Sichuan showed low tick activity and low concern (low–low cluster). The low-high cluster included Jiangsu, Zhejiang, Shanghai, and Jiangxi, where public concern was high despite lower tick occurrence. Conversely, the high-low cluster in the northwest, particularly Xinjiang Uygur Autonomous Region, highlighted areas of high tick activity but low public concern, indicating potential gaps in awareness or surveillance. These spatial patterns, visualized in Fig. 3 (and further mapped in Supplemental Figure S2), underscore the misalignment between tick-borne disease risk and public awareness, suggesting that targeted public health initiatives and educational outreach may be needed in regions with significant discrepancies.

## 4 Discussion

The results of this study provide significant insights into the public's concerns about ticks and tick-borne diseases in China, as reflected by trends in online discourse. The findings reveal a clear seasonal pattern in public interest, with a peak in April, coinciding with the onset of tick activity in many parts of the country [[13], [14], [15]]. This indicates a heightened awareness of the risks posed by ticks during this period, which corresponds with the typical tick lifecycle and an increase in outdoor human activities.

The distribution of posts related to ticks varies significantly across online platforms (Table 1). WeChat and Weibo emerge as the primary venues for discussions on tick biology and public awareness, indicating their central role in this discourse. In contrast, Network Video platforms show limited engagement with tick-related content, suggesting that such topics may not be well-suited for visual formats. When examining the keyword “tick bites,” WeChat continues to dominate, followed by Weibo and News platforms, while Network Video platforms demonstrate even lower engagement levels. This pattern highlights the challenges of sharing graphic content on visually-oriented media. For broader health topics like “Lyme disease” and “tick-borne encephalitis,” discussions are again concentrated on WeChat and Weibo, suggesting that, despite their importance, these health issues receive limited visibility outside social media channels. Overall, the variation in post distribution underscores the necessity of understanding platform-specific dynamics. The higher engagement on WeChat and Weibo reflects their popularity for social interaction, whereas lower activity on platforms like Network Video may result from content format preferences and moderation policies.

In comparing the spatial pattern of public concern with tick species distribution [11,12], there is some alignment, particularly in regions like South China (e.g., Fujian), where both high tick diversity and public discourse are prominent. However, notable disparities exist, such as in Xinjiang Uyghur Autonomous Region and Inner Mongolia, which host a significant number of tick species but show lower public concern, likely due to reduced awareness or media coverage. Conversely, regions like Zhejiang, Jiangsu and Shanghai exhibit elevated public concern on ticks and tick bites despite not being identified as tick diversity hotspots, suggesting that public awareness is influenced by factors beyond tick species abundance. Urbanization, socioeconomic development, and higher internet penetration in these areas may drive greater media coverage and public health campaigns, raising awareness of ticks even in regions with fewer species. Cultural and educational factors may also contribute to increased public interest. These discrepancies underscore the need for targeted awareness efforts in regions with high tick diversity but low public attention.

Both Lyme disease and tick-borne encephalitis show higher disease prevalence in northern and northeastern regions (Heilongjiang, Jilin, Inner Mongolia, and Xinjiang Uyghur Autonomous Region) [16]. However, public concern, as reflected in online discourse, is more concentrated in southern provinces like Zhejiang, Guangdong, and Jiangsu, where tick diversity may be lower but public awareness is elevated. Posts related to Lyme disease were distributed across both northern and southern provinces, indicating that public concern is not restricted to areas traditionally associated with tick-borne diseases. In contrast, posts related to tick-borne encephalitis were primarily concentrated in southern provinces such as Guangdong, which is inconsistent with the known geographic distribution of the disease [17]. This discrepancy may be due to an expansion of tick populations into previously unaffected areas or growing national awareness of Lyme disease, potentially influenced by increased media coverage or public health campaigns.

The focus of public concern on southern regions indicates that awareness is likely influenced more by factors such as media attention, public health initiatives, and healthcare infrastructure than by the actual burden or risk of the diseases. Several reasons may account for the concentration of posts in these areas. First, the prevalence of ticks is notably low in many southern provinces of China, suggesting a deficiency in tick-related research. Second, southern China generally benefits from more advanced information technology compared to the north, which facilitates the initiation and dissemination of media coverage in these regions. Third, limited internet access in certain northern areas likely exacerbates the disparity in post activity [18]. Additionally, it is important to note that posts related to other tick-borne diseases, such as severe fever with thrombocytopenia syndrome [19] and others [20], which have established distribution in southern regions, have not been included in this study due to their low volume of posts during 2023–2024. Moreover, tourism may play a role in shaping public awareness [21]. Travelers from south China to northeast China might be more aware of tick-borne disease risks due to travel advisories or personal experiences, contributing to heightened concern both before and after their trips. Consequently, there is an urgent need to enhance public awareness and disease education in northeastern and northwestern China, where the risk of both Lyme disease and tick-borne encephalitis is elevated, yet public concern remains comparatively low.

The correlation between online public interest and the known ecology of ticks and tick-borne diseases in China demonstrates the potential of leveraging big data from platforms such as People Cloud to monitor public health concerns in real-time. Such data could serve as an early indicator of tick activity and disease outbreaks, providing public health authorities with crucial insights to inform prevention strategies. The results also highlight significant gaps in public awareness regarding tick-borne diseases. Regions with high disease risk, such as northwestern and northeastern China, display lower public concern, as reflected in keyword search volumes. This finding underscores the need for focused educational efforts in areas with high disease risk but limited public engagement, to reduce the potential for outbreaks and improve disease prevention.

Despite the valuable insights, this study has several limitations. First, the data come exclusively from the People Cloud platform, which may not capture the concerns of individuals without internet access or those using other platforms and languages besides Chinese. Second, while efforts were made to filter out unreliable content, the variability in post quality, such as bias or incompleteness, may still affect the interpretation of public concern. Third, the relatively short timeframe of data collection represents another limitation; extending this period in future research could yield a more comprehensive understanding of long-term trends in public concern. Lastly, the study did not assess the content of posts in terms of sentiment or misinformation, which could offer deeper insights into the nature and accuracy of public concern.

## 5 Conclusion

This study reveals seasonal peaks in public concern about ticks and tick-borne diseases in China, aligned with tick activity. Geographic mismatches between tick distribution and public awareness suggest media and urbanization shape perceptions more than disease risk. Big online opinion data offers a valuable tool for real-time health monitoring, enabling early detection of outbreaks and guiding targeted prevention, especially in high-risk, low-awareness regions. Awareness gaps remain, particularly in northwestern and northeastern China, highlighting the need for focused educational efforts. Future research should expand data sources, assess sentiment and misinformation, and evaluate health interventions to optimize big data's role in managing tick-borne diseases.

## CRediT authorship contribution statement

**Yuxin Li:** Writing – original draft, Visualization, Software, Methodology, Investigation, Formal analysis, Data curation, Conceptualization. **Tengfei Hu:** Validation, Resources, Investigation, Data curation. **Tingting Wang:** Writing – review & editing, Visualization, Validation, Resources, Investigation. **Sen Li:** Writing – review & editing, Supervision, Resources, Funding acquisition, Conceptualization.

## Ethical considerations

The data utilized in this study were anonymized and publicly accessible through the People Cloud platform. No personally identifiable information (PII) was collected during the research process. Since the study relied on aggregated public data and did not involve human subjects, formal ethical approval was not required.

## Funding statement

This study was supported by the 10.13039/501100001809National Natural Science Foundation of China (grant number 42107458).

## Declaration of competing interest

The authors declare that they have no known competing financial interests or personal relationships that could have appeared to influence the work reported in this paper.
