# Supplementary material for: Mismatched online public concern and tick-borne disease risk in China
Source: Sci One Health. 2024 Dec 12;4:100101. doi: 10.1016/j.soh.2024.100101 (PMC11804818; doi:10.1016/j.soh.2024.100101)
Supplement: Multimedia component 1 [file mmc1.docx]

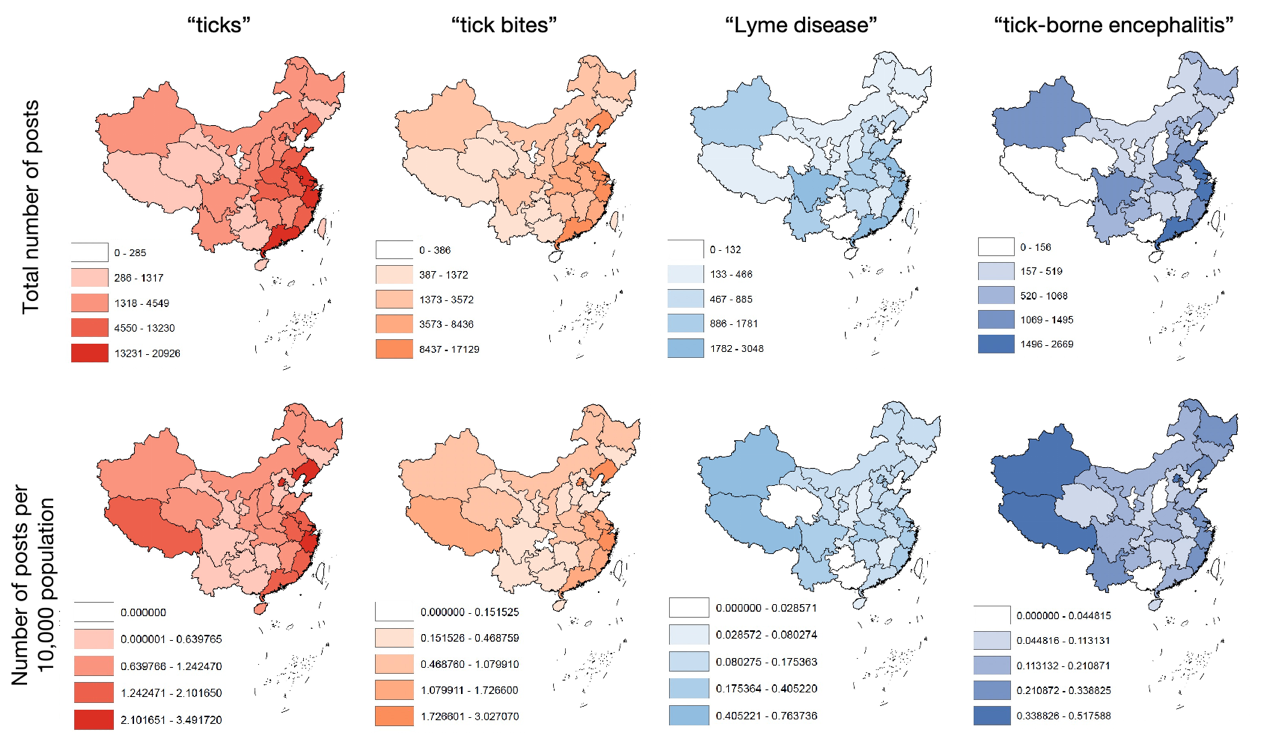


**Figure S1** Geographic distribution of the total number of tick-related online posts and the number of posts per 10,000 population across provincial-level administrative regions in China, illustrating regional variations in public engagement with tick-borne diseases.


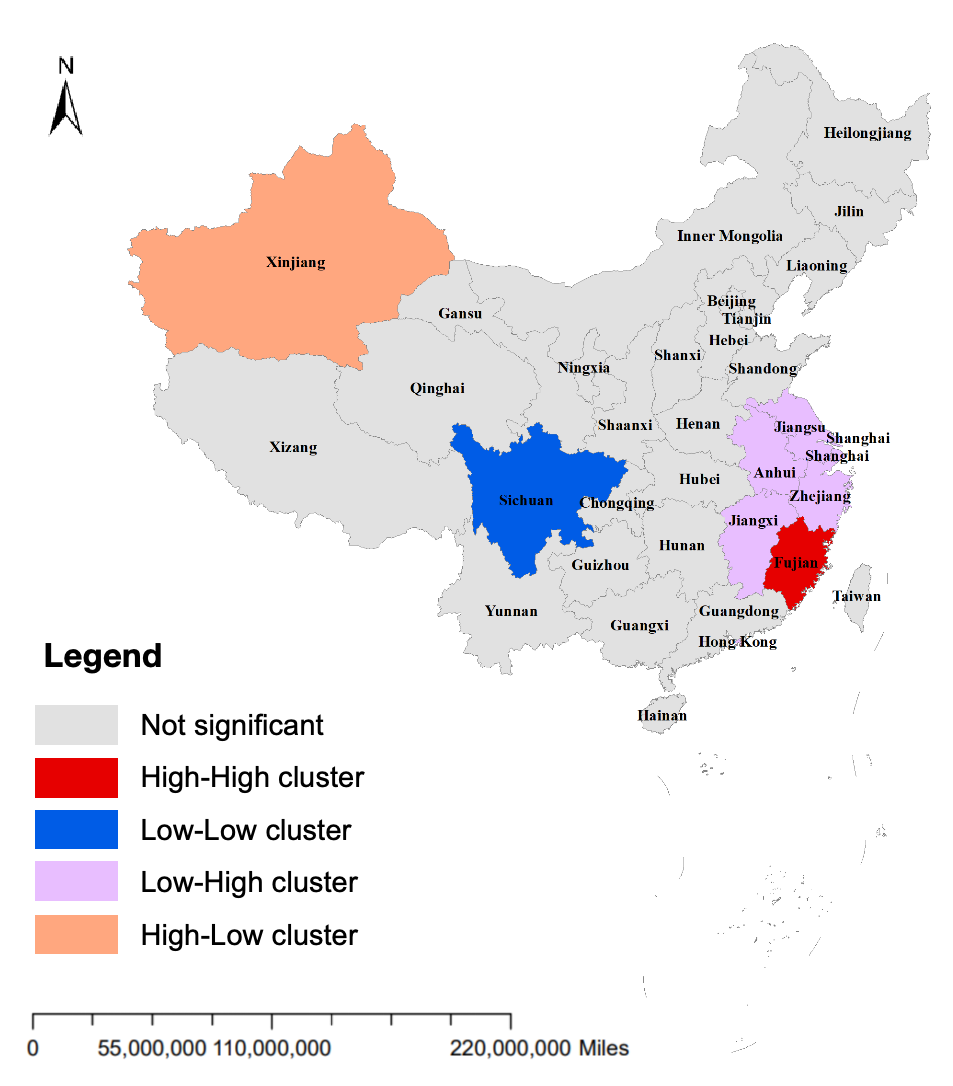


**Figure S2** Geographic distribution of bivariate Moran’s I results, illustrating spatial autocorrelation between tick-related online posts and tick occurrence records across regions in China.
